# Efficient Deployment of Multi-UAVs in Massively Crowded Events †

**DOI:** 10.3390/s18113640

**Published:** 2018-10-26

**Authors:** Ahmad Sawalmeh, Noor Shamsiah Othman, Hazim Shakhatreh

**Affiliations:** 1 Department of Electronics and Communications Engineering, Universiti Tenaga Nasional, 43000 Selangor, Malaysia; Shamsiah@uniten.edu.my; 2 Department of Telecommunications Engineering, Yarmouk University, 21163 Irbid, Jordan; hazim.s@yu.edu.jo

**Keywords:** Unmanned Aerial Vehicles (UAVs), path loss model, Particle Swarm Optimization (PSO), K-means algorithm, ternary search algorithm, Circle Packing Theory (CPT)

## Abstract

In this paper, the efficient 3D placement of UAV as an aerial base station in providing wireless coverage for users in a small and large coverage area is investigated. In the case of providing wireless coverage for outdoor and indoor users in a small area, the Particle Swarm Optimization (PSO) and K-means with Ternary Search (KTS) algorithms are invoked to find an efficient 3D location of a single UAV with the objective of minimizing its required transmit power. It was observed that a single UAV at the 3D location found using the PSO algorithm requires less transmit power, by a factor of 1/5 compared to that when using the KTS algorithm. In the case of providing wireless coverage for users in three different shapes of a large coverage area, namely square, rectangle and circular regions, the problems of finding an efficient placement of multiple UAVs equipped with a directional antenna are formulated with the objective to maximize the coverage area and coverage density using the Circle Packing Theory (CPT). Then, the UAV efficient altitude placement is formulated with the objective of minimizing its required transmit power. It is observed that the large number of UAVs does not necessarily result in the maximum coverage density. Based on the simulation results, the deployment of 16, 19 and 26 UAVs is capable of providing the maximum coverage density of 78.5%, 82.5% and 80.3% for the case of a square region with the dimensions of 2 km × 2 km, a rectangle region with the dimensions of 6 km × 1.8 km and a circular region with the radius of 1.125 km, respectively. These observations are obtained when the UAVs are located at the optimum altitude, where the required transmit power for each UAV is reasonably small.

## 1. Introduction

Unmanned Aerial Vehicles (UAVs) have increasingly diverse and ubiquitous roles in today’s society. They have been deployed in many civilian and military applications [1,2,3]. UAV can be used as an aerial base station and as a supplement to the existing ground base station when the cellular network is overloaded during a massively crowded special event or when the infrastructure of the terrestrial base stations is damaged due to natural disasters [4,5].

Al-Hajj (pilgrimage to Mecca) is an annual massively crowded event for the Muslims around the world. Every year, millions of pilgrims travel to the city of Mecca from all over the world for this event. During the peak of the event, more than two million pilgrims perform a series of rituals in Mecca city within a one-week duration. The movement of a huge number of pilgrims occurred at the four holy sites of Mina, Arafat, Mozdalifah and the holy Masjid Al-Haram. As a result of the huge gathering of pilgrims, where large demand for communication is required, terrestrial base station overloading may happen, and sometimes, the ground base station could not provide simultaneous coverage to all pilgrims in this event [6,7]. Consequently, instead of building a cellular infrastructure network for temporary crowded events [8], UAVs can be utilized as a supplement to the terrestrial base station, in providing uninterrupted coverage [9,10,11,12,13].

Some of the key technical challenges in the deployment of UAVs as aerial base stations are the efficient 3D deployment of UAVs, power consumptions, wireless coverage optimization and interference management [3,10,14,15]. There has been an increase of research interest in the efficient 3D deployment of UAV strategies. This is because the UAV deployment problem has an impact on the power consumptions [3,12,16]. Thus, this motivates research interest in the efficient 3D deployment of UAV strategies that aim for minimum transmit power.

### 1.1. Related Works

The efficient 3D deployment of UAVs strategies can be categorized into two categories, namely providing wireless coverage for outdoor users [9,10,11,13,14] and providing wireless coverage for indoor users [16,17].

Two issues that must be taken into consideration in the deployment of UAV as an aerial base station are the relation between the coverage area and the altitude of UAV and the effects of urban environment on the performance of communications.

There are two effects of the UAV placement, namely the coverage performance in terms of the number of users within the coverage area, as well as the quality of the Air-To-Ground (ATG) links. ATG communication occurs in accordance to two main propagation groups, namely, Line-Of-Sight (LOS) and Non-Line-Of-Sight (NLOS) conditions.

Several ATG channel models in a dense urban area have been studied in [9,18,19]. As presented in [20], the probability of LOS for ATG communication is a function of elevation angle and the average height of buildings. Furthermore, the characteristics of the ATG channel depends on the height of the aerial base stations due to the path loss and shadowing effects of obstacles, as discussed in [19].

In [9], the authors provided a generic statistical propagation radio model for predicting the ATG path loss between a Low Altitude Platform (LAP) and its corresponding ground terminal for LOS and NLOS connections based on statistical parameters for different environments of the target area. Furthermore, both issues were addressed in [9], namely the impact of the UAV placements on the number of users within the coverage area, as well as the effect of the environment on the occurrence of LOS. Many researches employ this path-loss model in addressing the UAV deployment problem [10,11,12,13]. However, the ATG path loss model [9] is suitable for outdoor ground terminals only.

The authors in [10] studied the impact of UAV’s altitude position on the coverage performance in terms of coverage area and minimum transmit power. More specifically, the ATG path loss model developed by Al-Hourani et al. [9] was utilized to find the optimal altitude of UAV that maximized the UAV coverage and minimized its required transmit power.

The authors in [11] utilized the ATG model to find the optimal deployment of UAVs acting as flying base stations to minimize the total required UAV transmit power while satisfying the users’ data rate requirements. In [12], the authors used this model to find the efficient UAV placement that maximized the network throughput. In [13], the study utilized this model to find the minimum number of UAVs and their 3D placement so that all the users were served. However, all previous studies assumed that all receivers are outdoors and located at 2D points. These assumptions limit the applicability of this model when we need to consider the indoor users.

Thus, a suitable path loss model needs to be studied, in the case of providing wireless coverage for indoor users. The authors in [17] utilized the outdoor-to-indoor path loss model certified by the International Telecommunication Union (ITU) [21] to provide coverage for indoor receivers. More specifically, in [17], the authors proposed an efficient 3D UAV placement that minimized the total transmit power required to provide wireless coverage for all indoor receivers, where the indoor receivers are symmetrically distributed.

In [3], the authors proposed an efficient 3D deployment of UAV using the Particle Swarm Optimization algorithm (PSO) to find an efficient 3D UAV location that minimized the total transmit power required to cover all indoor receivers where the indoor receivers were uniformly distributed inside the building. Due to the limited UAV transmit power, the authors in [16] minimized the number of UAVs required to cover the indoor receivers. However, the studies in [3,16,17] assumed that all receivers were located indoors. Furthermore, the above-mentioned strategies considered the 3D location of a single UAV and covering a small area of communication. As an example, in [3], it is assumed that all receivers are located in a building with the dimensions: horizontal building width $x_{b}$ is 20 m; vertical building width $y_{b}$ is 50 m; building heights are 200 m, 250 m and 300 m.

Thus, when considering providing wireless coverage over a larger coverage area, this leads to concerns about the number of UAVs that need to be employed. In this situation, the UAV deployment strategy that maximizes the coverage area can be used. In [14], the authors utilized Circle Packing Theory (CPT) to find an efficient placement of multiple UAVs acting as aerial base stations that maximizes the coverage area. However, this method is appropriate only for a circular coverage area.

Explicitly, this paper extends the work in [22] by proposing the UAV deployment strategy for a large coverage area. More specifically, in this paper, an efficient 3D deployment strategy is presented to find an efficient 3D placement of a single UAV that minimizes the transmit power. The performance of the 3D location of the UAV is analyzed in terms of the required transmit power when using two different algorithms, namely the PSO and K-means with Ternary Search (KTS) algorithms. Next, to provide wireless coverage over a large area, a 3D deployment strategy is proposed in a way that the total coverage over a large area is maximized using CPT. Thus, this leads to finding the number of UAVs equipped with a directional antenna needed to cover the area. The coverage performance is analyzed for three different shapes of area, namely square, rectangle and circular, in terms of coverage density.

### 1.2. Paper Contributions

The contributions of this paper are summarized as follows:

- The existing ATG path loss model [9] and outdoor to indoor path loss model [21] are used to study the problem of a single UAV placement to provide coverage in crowded events for both outdoor and indoor receivers simultaneously, with the objective to minimize the required UAV transmit power. Due to the intractability of the formulated problem, two algorithms are developed to find an efficient 3D UAV placement using two optimization techniques, namely the PSO and KTS algorithms. The proposed algorithms consider the problem in providing wireless coverage for indoor and outdoor users, in a small area using a single UAV.
- The efficient 3D placements of multiple UAVs that provide maximum wireless coverage and minimize the transmission power are found for each UAV.
- The CPT is utilized to find the number of UAVs needed for providing wireless coverage for outdoor users in a large coverage area having three different shapes of coverage area, namely square, rectangle and circular. The problem is formulated with the objective to maximize the wireless coverage area using multiple UAVs. In each subarea, the UAV altitude is optimized using the algorithm to provide wireless coverage using a single UAV above.

The rest of this paper is organized as follows. In Section 2, the case of providing wireless coverage for outdoor and indoor users using a single UAV is studied, where the objective is to minimize the UAV transmit power. Section 3 presents the study of the problem in providing wireless coverage using multiple UAVs equipped with directional antennas, where the objective is to maximize the wireless coverage over a large area. Three different shapes of 2D region are considered, with each UAV employing a directional antenna, which produces a circular coverage pattern. Each circular cell is referred to as a subarea. Section 4 presents the simulation results and analysis for the deployment of the single UAV and multiple UAVs scenarios. Section 4.3 discusses the main observations obtained from the simulation results. The conclusion and future work are presented in Section 5.

## 2. Providing Wireless Coverage Using a Single UAV

### 2.1. System Model

**Figure 1:**
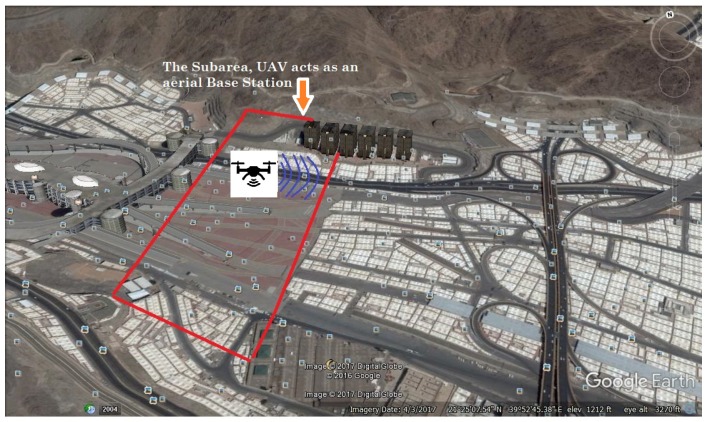
Providing wireless coverage using a single UAV.

Consider a coverage area that is divided into *K* subareas. In each subarea, there are *N* outdoor receivers and *M* indoor receivers, which are non-uniformly distributed using a beta random distribution, denoted as function f(x,y). Let [$x_{1}$,$y_{1}$] × [$x_{2}$,$y_{2}$] denote the dimension of the subarea. Then, the location of each outdoor receiver $i_{out}$ is denoted by ($x_{i_{out}}$, $y_{i_{out}}$, 0); the location of indoor receiver $i_{in}$ is denoted by ($x_{i_{in}}$, $y_{i_{in}}$, $z_{i_{in}}$); and the 3D UAV location is represented by ($x_{UAV}$, $y_{UAV}$, $z_{UAV}$). In this model, it is considered that a single UAV serves as an aerial base station, as shown in Figure 1.

#### 2.1.1. ATG Path Loss Model

In this system, the ATG path loss prediction between an LAP and ground terminals presented in [9,23] is utilized. More specifically, the ATG path loss is modeled by considering the probability of Line Of Sight (LOS) and Non-Line Of Sight (NLOS) links, which depend on the urban environment parameters, building heights and elevation angle. The probability of LOS and NLOS links can be given as [9]:

(1) $$\begin{array}{c} P_{LOS}=\frac{1}{1+a.exp(-b\left[\frac{180}{\pi }\theta_{out}-a\right])} \end{array}$$

(2) $$\begin{array}{c} P_{NLOS}=1-P_{LOS} \end{array}$$

where *a* and *b* are constant values that depend on the environment; such as urban, suburban, dense urban and rural environments; whilst the elevation angle $\theta_{out}$ is given as $\theta_{out}$ = $\sin^{-1}\left(h/d\right)$, where the distance between UAV and the ground receiver is *d*=$\sqrt{h^{2}+r_{d}^{2}}$ and *h* is the UAV altitude. $r_{d}$ denotes the distance between UAV projection at the xy plane ($x_{UAV},y_{UAV}$) and the ground receiver coordinates located at ($x_{i}$,$y_{i}$), which is given as $r_{d}$=$\sqrt{{\left(x_{UAV}-x_{i}\right)}^{2}+{\left(y_{UAV}-y_{i}\right)}^{2}}$. The average path loss $\bar{L}$ (dB) for LOS and NLOS links can be formulated as [9]:

(3) $$\begin{array}{c} \bar{L}\left(dB\right)=P_{LOS}\times L_{LOS}+P_{NLOS}\times L_{NLOS} \end{array}$$

where the path loss for the LOS link, $L_{LOS}$, and the path loss for the NLOS link, $L_{NLOS}$, are given as:

$$\begin{array}{c} L_{LOS}=20\log\left(\frac{4\pi f_{c}d}{c}\right)+\eta_{LOS}\\ L_{NLOS}=20\log\left(\frac{4\pi f_{c}d}{c}\right)+\eta_{NLOS} \end{array}$$

where $f_{c}$ is the carrier frequency, *c* is the speed of the light, whilst $\eta_{LOS}$ and $\eta_{NLOS}$ are additional losses, which depend on the environment for LOS and NLOS links, respectively.

#### 2.1.2. Outdoor-to-Indoor Path Loss Model

Furthermore, in order to provide the wireless coverage for indoor users, the path loss model for outdoor-to-indoor certified by ITU is utilized. The path loss is given as [21]:

(4) $$\begin{array}{c} L_{i}\left(dB\right)=L_{FSP}+L_{BP}+L_{IN} \end{array}$$

where $L_{FSP}$ is the free space path loss, $L_{BP}$ is the penetration loss for the building and $L_{IN}$ is the loss inside the building.

The free space path loss $L_{FSP}$ is given as $L_{FSP}$ = $20log(d_{3D})$ + $20log(f_{c(GHz)})$ + 32.4, where $d_{3D}$ is the distance between an indoor receiver $i_{in}$ and the UAV, and the carrier frequency $f_{c(GHz)}$ is equal to 2 GHz.

The building penetration loss $L_{BP}$ is given as $L_{BP}$ = 14 + 15 ${\left(1-cos\theta_{in}\right)}^{2}$, where the incident angle is denoted as $\theta_{in}$ and the loss inside the building $L_{IN}$ is given by $L_{IN}$ = 0.5$d_{2D_{in}}$, where $d_{2D_{in}}$ is the 2D indoor distance between UAV and receiver $i_{in}$ located at ($x_{i_{in}}$, $y_{i_{in}}$, $z_{i_{in}}$). More specifically, $d_{2D_{in}}$ denotes the indoor part of the 2D distance between the UAV and the indoor receiver, $i_{in}$.

The building penetration loss $L_{BP}$ depends on the altitude of the indoor receiver $z_{i_{in}}$; whilst the free space path loss $L_{FSP}$ is related to the distance between an indoor receiver $i_{in}$ and the UAV $d_{3D}$, which depends on the altitude of the indoor receiver $z_{i_{in}}$. Thus, the altitude of the indoor receiver $z_{i_{in}}$ affects the path loss in terms of $L_{FSP}$ and $L_{BP}$.

### 2.2. Problem Formulation

Consider a single UAV that is located at ($x_{UAV}$, $y_{UAV}$, $z_{UAV}$) transmitting data to the outdoor receiver $i_{out}$ located at ($x_{i_{out}}$, $y_{i_{out}}$, 0) or to the indoor receiver $i_{in}$ located at ($x_{i_{in}}$, $y_{i_{in}}$, $z_{i_{in}}$). The data rate for each receiver *i* that is located either indoors or outdoors is given by:

(5) $$\begin{array}{c} r_{i}=Clog_{2}\left(1+\frac{\left(\frac{P_{t,i}}{L_{i}}\right)}{Np}\right) \end{array}$$

where *C* is the receiver bandwidth, $P_{t,i}$ is the transmit power of UAV for receiver *i*, $L_{i}$ is the path loss between UAV and receiver, *i*, and Np is the noise power. In this work, the interference is implicitly modeled as noise.

In this system model, Frequency Division Multiple Access (FDMA) is used as the channel access technique. It is assumed that each UAV allocates equal channel bandwidth to receivers, and in order to avoid interference, each channel is assigned to one receiver.

In this problem, it is considered that the UAV transmits data to *N* outdoor and *M* indoor receivers at a desired data rate (*r*). (*M* + *N*) is the total number of receivers inside the coverage subarea, and each receiver has a channel with the bandwidth equal to *A*/(M+N), where *A* is the transmission bandwidth of an UAV. The total required transmit power of UAV to satisfy the data rate *r* for all receivers can be formulated as:

(6) $$\begin{array}{c} P=\sum_{i=1}^{\left(M+N\right)}\left(2^{\frac{r(M+N)}{A}}-1\right)NpL_{i} \end{array}$$

The objective is to find an efficient 3D placement of UAV, such that the total required transmit power of UAV is minimized, and at the same time, the desired data rate of all receivers is satisfied. However, the optimal UAV 3D placement that minimizes the total transmit power becomes complicated, as the optimal solutions can be obtained by searching all possible sets of solutions [13], while satisfying the data rate for all receivers. Thus, the efficient solution can be found using the meta-heuristic algorithm.

### 2.3. Efficient UAV 3D Placement Algorithms

Due to the intractability of this problem, two algorithms are proposed to find an efficient 3D placement of a single UAV using two optimization techniques, namely the PSO and KTS algorithms. In PSO, a member of the set of possible solutions is known as a candidate solution, which is referred to as a particle. PSO utilizes the number of particles, and each particle moves around in the search space looking for the best solution. On the other hand, KTS includes two algorithms, namely the K-means algorithm and the Ternary Search (TS) algorithm. K-means is a clustering algorithm that attempts to split a given dataset into a fixed number of *k* clusters; whilst the ternary search employs the divide and conquer algorithm used to find the minimum or maximum values of a function.

#### 2.3.1. Particle Swarm Optimization Algorithm (PSO)

In 1995, the PSO algorithm was introduced by Kennedy and Eberhart [24]. PSO is an intelligent optimization algorithm that is based on the swarm intelligence paradigm inspired by animals’ social behavior such as a flock of birds or a school of fish. In PSO, the optimization problem is solved by iteratively trying to improve a candidate solution based on the local best experience for each particle and the global best experience for all candidates. In PSO, every particle is a candidate solution to the optimization problem.

Algorithm 1 shows the pseudocode of the proposed algorithm using the PSO optimization technique. Step 1 in Algorithm 1 presents the inputs of the algorithm, namely N_pop defines the population of candidate solutions of the algorithm, *W* refers to the inertia weight, while $r_{1}$ and $r_{2}$ denote the two random numbers uniformly distributed randomly in a range between zero and one and $c_{1}$ and $c_{2}$ are the acceleration coefficients.

In the initialization step, the values of the constriction factor, κ, cognitive parameter, $\phi_{1}$, and the social parameter, $\phi_{2}$, must be selected, where κ = 1 and $\phi_{1}$ + $\phi_{2}$ > 4 [25]. This will help the PSO to find the efficient solution for the optimization problem.

The PSO is initialized with a group of random solutions for all particles’ positions, *Lo*, and the particles’ velocity, *V*, as shown in Steps 7 to 14. Then, in every iteration, for each particle, the best local location, *L_BL*, and the velocity are updated according to Equations (7) and (8), respectively. Moreover, the global best location, *G_BL*, is also updated, as in Steps 15 to 25. This will help the swarm of particles to move toward the best solution.

(7) $$\begin{array}{c} Lo_{i}\left(t+1\right)=Lo_{i}\left(t\right)+V_{i}\left(t+1\right) \end{array}$$

(8) $$\begin{array}{c} V_{i}\left(t+1\right)=W*V_{i}\left(t\right)+r1*c1*\left(L\_{BL}_{i}\left(t\right)-Lo_{i}\left(t\right)\right)+r2*c2*\left(G\_BL\left(t\right)-Lo_{i}\left(t\right)\right) \end{array}$$

#### 2.3.2. K-Means with Ternary Search Algorithms

K-means is an algorithm that uses an iterative refinement technique to solve the clustering problem. This is accomplished by partitioning a given dataset into *k* clusters. In this algorithm, each cluster is represented by its centroid. Thus, there are *k* centroids for *k* clusters. Each point in the dataset is assigned to a cluster that has the nearest centroid. More specifically, the K-means algorithm can be performed in the following stages:

1. Initially, random guesses for cluster centroids are made, as shown in Step 3 of Algorithm 2.
2. The nearest centroid is determined for each data point, by calculating the Euclidean distance between each point and the centroid of the cluster, as shown in Step 5 of Algorithm 2.
3. In each cluster, the centroid is replaced by a new value. This new value is the means of the points belonging to the cluster, as in Step 6 of Algorithm 2.
4. Repeat the process in Items 2 and 3 above, until the solution converges. The convergence happens when the centroids and their locations are no longer changed; more specifically, when the cluster mean is not changed as in Steps 4 to 6 of Algorithm 2.

**Algorithm 1** Particle swarm optimization algorithm.
1: **Input:****Vmin**: Lower bound decision variable.**Vmax**: Upper bound decision variable.$c_{1}$**and**$c_{2}$: Acceleration coefficients.$r_{1}$, $r_{2}$: Uniformly-distributed random number U(0, 1).N_it: Number of iterations.N_pop: Population size.(κ, $\phi_{1}$, $\phi_{2}$): Construction coefficients.2: **Initialization:**3: ϕ = $\phi_{1}$ + $\phi_{1}$,4: χ = $2\kappa /|2-\phi -{\left(\phi^{2}-4\phi \right)}^{0.5}|$5: W = χ, c1 = χ$\phi_{1}$, c2 = χ$\phi_{2}$,6: globalbest.cost = ∞7: **for** i = 1:N_pop8: Lo${}_{i}$(*t*) = unifrnd(Vmin, Vmax, Vsize)9: V${}_{i}$(*t*) = zeros(Vsize)10: cost${}_{i}$ = costfunction(Lo${}_{i}$)11: best.Lo${}_{i}$(*t*) = Lo${}_{i}$(*t*)12: best.cost${}_{i}$(*t*) = cost${}_{i}$(*t*)13: **if** best.cost${}_{i}$(*t*) < globalbest.cost14:  globalbest = best_cost${}_{i}$(*t*) **end if**
**end**
15: **PSO Loop:**16: **for** t = 1:N_it17: **for**i = 1:N_pop18: V${}_{i}$(*t* + 1) = W * V${}_{i}$(*t*) + c${}_{1}$ * r${}_{1}$ .* Lo${}_{i}$(*t*)) + c${}_{2}$ * r${}_{2}$ .* (best.Lo${}_{i}$(*t*) − (globalbest_Lo - Lo${}_{i}$(*t*))19: Lo${}_{i}$(*t* + 1) = Lo${}_{i}$(*t*) + V${}_{i}$(*t* + 1)20: cost${}_{i}$(*t*) = costfunction(Lo${}_{i}$(*t*))21:  **if** cost${}_{i}$(*t*) < best_cost${}_{i}$(*t*)22:  best_Lo${}_{i}$(*t*) = Lo${}_{i}$(*t*)23:  best_cost${}_{i}$(*t*) = cost${}_{i}$(*t*)24:  **if** best_cost(*t*) < globalbest.cost25:   globalbest = best_cost${}_{i}$(*t*)  **end if** **end if**  **end**
**end**



Moreover, the TS algorithm is used in tandem with the K-means algorithm, to determine the position of a specific value in a dataset. The sorted dataset is divided into three parts, and after that, the ternary search determines in which part the element exists. In Algorithm 2, Step 7 initiates the min and max variables for the left and right edge of the intervals, respectively. Then, in Step 10, the TS algorithm will repeat Steps 11 to 16 while max−min<ϵ. In this work, K-means is used to find the 2D placement ($x_{UAV}$,$y_{UAV}$) of UAV, since the outdoor and indoor receivers in the coverage area are assumed to be non-uniformly distributed. Then, the ternary search algorithm is used to find the UAV altitude ($z_{UAV}$). Algorithm 2 shows the pseudocode of the KTS algorithm.

**Algorithm 2** K-means with ternary search algorithm.

**1-K-means algorithm:**
 1: **Input:**  *K*: number of clusters, $x_{i}$: data points *i* = 1…n, $c_{k}$: set of centers *k* = 1…K,  $u_{k}$: cluster position that minimizes the distance from the data points to the cluster, *k* = 1…K 2: **Initialization:** 3: $c_{i}$ = random(num) 4: **Loop until convergence ∀*j* = 1:*n*** 5:
$$\sum_{k=1}^{K}\sum_{i\in c_{k}}d\left(x-u_{k}\right)=\sum_{k=1}^{K}\sum_{i\in c_{k}}\left|\right|x_{i}-u_{k}{\left|\right|}^{2}$$  $x_{i}$ = *j*: $d(x_{j},u_{i})$≤$d(x_{j},u_{l}$), *l*≠*i* 6: $u_{i}$ = $\frac{1}{c_{i}}$$\sum_{j\in c_{k}}X_{j},\forall i$ **end**
**2-Ternary Search Algorithm:**
 7: **Input:** *a*: interval left edge, *b*: interval right edge, *f*: function, ϵ: tolerance. 8: **Initialization:** 9: *l* = *a*, *r* = *b*. 10: **while** (r−l>ϵ) 11: x1 = (2∗l + *r*) / 3. 12: x2 = (*l* + 2∗r) / 3. 13: if f(x1)<f(x2) 14: l=x1 15: else 16: r=x2 17: return *r* **end**


## 3. Providing Wireless Coverage Using Multiple UAVs Equipped with Directional Antennas

In this section, an efficient deployment of multiple UAVs equipped with directional antennas is proposed. More specifically, the UAV deployment strategy to provide wireless coverage for receivers distributed over a large area of square, rectangle and circular 2D regions is investigated. Each UAV employs a directional antenna, which forms a subarea having a circular coverage pattern, referred to as the circle cell. Therefore, the CPT [26] is utilized to divide the total coverage area into non-overlapped circle cells, such that the coverage area is maximized. In order to avoid the interference between contiguous cells, the non-overlapped constraint is considered. Then, for each circle cell, an efficient 3D UAV placement that minimizes the total UAV transmit power required to cover all receivers within the cell is found. Furthermore, in this work, it is assumed that the distribution of the users is not given. Thus, the CPT is more suitable to be utilized in finding the UAV 2D placement, when compared with the K-means algorithm. Moreover, in this work, an efficient deployment of multiple UAVs is determined, such that the coverage area and density are maximized. However, the proposed algorithm presented in Section 2 does not consider this objective.

### 3.1. Case of a Square Region

Gatherings of people in some crowded events can form a square shape, such as the gathering of pilgrims in Arafat. The optimal packing of *n* identical and non-overlapped circles in a unit square is a widely-explored problem in the literature [27,28,29]. Many researchers have applied the CPT to find the maximum radius of *n* equal circles that can be packed in a unit square without overlapping.

In this scenario, it is assumed that the ground receivers are located in a geographical area that has a square shape and *n* UAVs can be deployed to maximize the total wireless coverage and density. More specifically, CPT is utilized to divide the total coverage area into non-overlapped circle cells. Then, for each cell, one UAV can be deployed to provide wireless coverage for receivers within the cell.

#### 3.1.1. Problem Formulation

The packing of equal circles in a square can be formulated in the following way:

- P1 : Place *n* identical non-overlapping circles in a unit square, with the objective function to maximize the radius of the circles *r*, such that the coverage area and coverage density are maximized.

The problem P1 is a continuous, nonlinear, inequality-constrained global optimization problem [30]. The problem P1 can be formulated as:

(9) $$\begin{array}{cc} \;\max_{x_{i},y_{i}}\;r\\ subject\;to:\\ \;r\leq x_{i}\leq 1-r, & \forall i\;\epsilon \;I=\left(1,....,n\right)\;\;.....\left(9.a\right)\\ \;r\leq y_{i}\leq 1-r, & \forall i\;\epsilon \;I=\left(1,....,n\right)\;\;.....\left(9.b\right)\\ \;\sqrt{{\left(x_{i}-x_{j}\right)}^{2}+{\left(y_{i}-y_{j}\right)}^{2}}\geq 2r, & \forall \;i\neq j\;\;.....\left(9.c\right)\\ \;\left(x_{i},y_{i}\right)\epsilon \left[0,1\right] & \forall i\in I=\left(1,....,n,\right)\;\;.....\left(9.d\right) \end{array}$$

where ($x_{i},y_{i}$) denotes the center coordinates of the circle *i*, $\sqrt{{\left(x_{i}-x_{j}\right)}^{2}+{\left(y_{i}-y_{j}\right)}^{2}}$ is the Euclidean distance between the centers of circles *i* and *j*, 1≤i<j≤n and ris the coverage radius of each cell. The objective function is to maximize the radius of the coverage cell. The constraint equations of (9.a) and (9.b) ensure that all packed circles lie inside the square. The constraint equation of (9.c) guarantees no overlapping between circles.

There has been a number of optimum solutions proposed for the problem of placing *n* identical non-overlapping circles in a unit square, such that the circle radius is maximized [27,28,29]. More specifically, in [27,28,29], the optimum arrangement of *n* identical non-overlapping circles in a unit square was solved by maximizing the distance *m* between any pairs of circles.

For a unit square, the density of packing *n* identical non-overlapping circles $d_{n}$ can be defined as the ratio of area occupied by the packed circles to a unit square area. Thus, the density of the packed circles in a unit square is related to the maximum radius of the packed circle $r_{n}$ by the following equation [31]:

(10) $$\begin{array}{c} d_{n}=nr_{n}^{2}\pi \end{array}$$

Table 1 shows the optimal results of the maximum radius of the packed circle $r_{n}$ and the corresponding maximum density $d_{n}$ of packing equal circles in a unit square for 2≤n≤22.

In this work, the coverage density of packing *n* identical non-overlapping circles $d_{n}$ is defined as the ratio of the area occupied by the packed circles to the area of the coverage region.

### 3.2. Case of a Rectangle Region

There are many crowded events where the users can form a rectangular shape, for example the movement of a huge number of attendees through streets or rectangular paths, such as the movement of pilgrims in Mina. The packing of *n* identical circles into a rectangle can be efficiently used to divide the rectangle region into equal circle cells, and for each cell, one UAV can be deployed to provide wireless coverage for users within the cell. This problem is known as an NP-hard problem, so finding the optimal packing solution is difficult and makes the problem very complicated [26,35,36]. Therefore, meta-heuristics approaches or local exhausted search methods can be used to solve this problem and find an efficient packing solution.

In [37], the authors proposed a heuristic algorithm to find the maximum radius of a specified number of non-overlapped equal circles inside a fixed size rectangle. They used the Formulation Space Search (FSS) approach to solve the circle packing problem.

#### 3.2.1. Problem Formulation

The problem of packing equal circles in a rectangle region can be formulated as follows:

- P2: Place *n* identical non-overlapping circles in a rectangle region L×W, with the Cartesian origin (0,0) as the rectangle center. The objective function is to maximize the radius of the circles *r* such that the coverage area and density are maximized.

In problem P2, $R_{overlap}$ is the upper bound radius of the packed circles, and it can be defined by $n\pi R_{overlap}$ = LW, then $R_{overlap}$ = $\sqrt{LW/n\pi }$. A mixed Cartesian and polar formulation has been used. Hence, problem P2 is a mixed nonlinear formulation and can be formulated as [37]:

(11) $$\begin{array}{c} \max_{x_{i},y_{i}}\;r\;\\ subject\;to:\;\\ -L/2\leq x_{i}+r\leq L/2;\;-W/2\leq y_{i}+r\leq W/2\;.........................\left(11.a\right)\\ -L/2\leq x_{i}-r\leq L/2;\;-W/2\leq y_{i}-r\leq W/2\;.........................\left(11.b\right)\\ \forall \;i\;\epsilon \;C:\;Cartesian\;Set\;\\ -L/2\leq r_{i}cos\left(\theta \right)+r\leq L/2;\;-W/2\leq r_{i}sin\left(\theta \right)+r\leq W/2\;\;....\left(11.c\right)\\ -L/2\leq r_{i}cos\left(\theta \right)-r\leq L/2;\;-W/2\leq r_{i}sin\left(\theta \right)-r\leq W/2\;....\left(11.d\right)\\ \forall \;i\;\epsilon \;P:\;Polar\;Set\;\\ 0\leq r_{i}\leq \sqrt{{\left(L/2\right)}^{2}+{\left(W/2\right)}^{2}},\;\forall \;i\;\epsilon \;P\;.........................\left(11.e\right)\\ \sqrt{x_{i}-x_{j}{)}^{2}+{\left(y_{i}-y_{j}\right)}^{2}}\geq 2r,\;\forall \;i\neq j\;.........................\left(11.f\right)\\ 1\leq i<j\leq n\;\\ 0\leq \theta \leq 2\pi \;.........................(11.g)\\ 0\leq r\leq R_{Overlapped}\;.........................\left(11.h\right), \end{array}$$

where ($x_{i},y_{i}$) is the circle *i* center. The first four constraints ensure that all packed circles are located inside the rectangle. $\sqrt{{\left(x_{i}-x_{j}\right)}^{2}+{\left(y_{i}-y_{j}\right)}^{2}}$ is the Euclidean distance between the centers of circles *i* and *j*, ∀1≤i<j≤n, and this distance must be at least 2r to ensure that there is no overlap between circles. The objective of this problem is to maximize the radius of *n* identical non-overlapping circle cells. The constraints of Equations (11.a),(11.b),(11.c) and(11.d) ensure that all packed circles are located inside the coverage area. Moreover, the constraints of Equations (11.e) and (11.f) guarantee no overlapping between cells.

Similarly, the coverage density of packing *n* identical non-overlapping circles $d_{n}$ is defined as the ratio of area occupied by the packed circles to the area of the coverage region, as discussed in Section 3.1.

#### 3.2.2. Algorithms for Packing Circles in a Rectangle Region

Many algorithms were developed for solving the problem of packing equal circles in a rectangle region, using meta-heuristic approaches [36,37,38,39,40]. In [36], two algorithms were developed using the meta-heuristic simulated annealing optimization technique to solve the packing problem of circular-based items into a rectangular pallet, such that the number of circular-based items being packed in the rectangular pallet was maximized without overlaps. These algorithms were referred to as the Cylinder Packing Algorithm using Minimizing Overlap with Simulated Annealing (CPA-MinOSA) and the Cylinder Packing Algorithm using Maximizing the Number of circles with Simulated Annealing (CPA-MaxNSA). The pseudocode of the CPA-MinOSA algorithm is shown in Algorithm 3.

In [37], a heuristic algorithm was proposed to solve the problem of packing *n* identical non-overlapping circles into a 2D container of fixed size, such that the circle radius was maximized. This algorithm was developed based on the Formulation Space Search (FSS) method. Algorithm 4 illustrates the FSS pseudocode.

Moreover, the Packomania website [40] used heuristic algorithms to find the best known solutions for packing identical circles in fixed size shapes, such that the circle radius and packing density were maximized.

**Algorithm 3** CPA-MinOSA algorithm.

**Input:**
n1=Number_of_circles.n2 = New_Number_of_Circles.Number_of_circles=Lower_bound() {Initial n1≤n2}.MinOSA: Minimizing overlap with simulated annealing.
**repeat**
 Result = MinOSA(n1); **if** overlap(n2) == 0 **then**  n2 = Result;  n1=n1+1 **end if****until** overlap(n2) ≠ 0


**Algorithm 4** Formulation space search pseudocode.

**Input:**
*Q*: Set of all pairs (i,j)ϵI,J=1,…,n, *C*: Set of circles in Cartesian coordinates.*P*: Set of circles in polar coordinates. $R_{best}$: Max. radius over all iterations.δ: Max. shift dist.of the circle center,$R_{Overlapped}$: Max. radius that circles can have before overlapping occurs. $C_{itr}$: Counter iteration.**Initialization**$\delta =\left(2/3\right)R_{Overlapped}$,
$R_{best}=0$
(X,Y) ←rnd(X,Y){Randomly generating *n* circles into plane}
$C_{itr}=0$

**iterative process:**

**repeat**
 $Q\leftarrow Overlapped\_Set(X,Y,\delta ,R_{Overlapped})$, (x,y)←$NLP(x_{0},y_{0},Q,C,P,X,Y,\delta ,R_{Overlapped})$ {Solve the non-linear optimization problem to give (x,y), $x_{0}\epsilon \left(X-\delta ,X+\delta \right)$, $y_{0}\epsilon \left(Y-\delta ,Y+\delta \right)$} $R_{new}$←$correction(x_{0},y_{0},x,y)$ {Radius correction [37]} $R_{best}$ = Max($R_{new},R_{best}$) {Update $R_{best}$} **if** $R_{new}<0.001$ **then**  δ = $\frac{1}{10}R_{Overlapped}$; **else**  $\delta =\frac{2}{3}R_{Overlapped}$ **end if** $C_{itr}$ = $C_{itr}+1$    {Update counter} C←P, P← {1,….,*n*} - *C*  {C & P Sets Swap} (X,Y) = (x,y) {(X,Y) ← current solution }**until** Reach termination condition


### 3.3. Case of a Circular Region

In this section, a circular coverage region is considered. The CPT of packing equal circles into a circular region is utilized, to provide wireless coverage using UAVs for receivers within the circular coverage region. Here, the problem to find the maximum radius of equal circles that can be packed into a unit circle with radius of r=1 is discussed. Moreover, the mathematical formulation and the main algorithm used to solve this problem is presented [37,41,42] in this section.

#### 3.3.1. Problem Formulation

The problem of packing equal circles into a unit circular region can be formulated as:

- P3: Place *n* identical non-overlapping circles into a unit circle with radius of r=1. The objective function is to maximize the radius of the packed circles, such that the coverage area and density are maximized.

The problem P3 can be formulated as:

(12) $$\begin{array}{cc} \;\max_{x_{i},y_{i}}\;r & \\ subject\;to: & \\ \;\sqrt{{\left(x_{i}-x_{j}\right)}^{2}+{\left(y_{i}-y_{j}\right)}^{2}}\geq 2r, & \forall \;i\neq j\;.......\left(12.a\right)\\ & 1\leq i<j\leq n\;\\ \;x_{i}^{2}+y_{i}^{2}\leq {\left(1-r\right)}^{2} & 1\leq n\leq n\;.......\left(12.b\right)\\ \;\left(x_{i},y_{i}\right)\;\epsilon \;\left[-1,1\right] & i=1,....,n\;.......\left(12.c\right) \end{array}$$

where ($x_{i},y_{i}$) is the center coordinates of a circle *i*, and the constraint equation of (12.a) is the Euclidean distance between the center of circles *i* and *j*, which is defined as $\sqrt{{\left(x_{i}-x_{j}\right)}^{2}+{\left(y_{i}-y_{j}\right)}^{2}}$ and must be ≥2*r* to ensure no overlapping between circles. The second constraint equation of (12.b) guarantees that the packed circles must fully lie inside the circular region.

Here, the coverage density of packing *n* identical non-overlapping circles $d_{n}$ is defined as the ratio of area occupied by the packed circles to the area of the coverage region, as discussed in Section 3.1.

#### 3.3.2. Algorithms for Packing Circles into a Circular Region

The authors in [37] presented the formulation of the circle packing problem inside a fixed size container with the objective to maximize the radius of the packed circles. To solve this problem, they used the FSS algorithm with the Sparse Nonlinear OPTimizer solver (SNOPT), as discussed in Section 3.2.2.

In [41], the authors found the densest packing of identical circles inside a unit circle using the general Reformulation Descent (RD) heuristic to maximize the radius of the packed circles into a fixed size container. They proposed nonlinear reformulation to transform one coordinate system to the other, such as switching from Cartesian to polar coordinates and vice versa. This solution was invoked in order to solve local search using the gradient method when it reached a stationary point in the Nonlinear Programming problem (NLP). It was observed that this technique allowed the NLP-solver to find a better solution. Moreover, the Packomania website [40] shows the best known solutions for packing *n* identical circles inside a unit circle with the maximum common circle radius and maximum packing density.

In addition to the previous convex shapes, CPT can be utilized in the problem of providing wireless coverage for crowded events that have other convex shapes such as an equilateral triangle [43] and a polygon [44]. Moreover, CPT can be also utilized for the problem of providing wireless coverage for crowded events that have non-convex 2D shapes [45].

## 4. Simulation Results and Analysis

### 4.1. Providing Wireless Coverage Using a Single UAV

This section presents the simulation results of the proposed algorithms to find an efficient 3D placement of a single UAV for providing wireless coverage for outdoor and indoor users, such that the transmit power is minimized.

In this simulation, an area in Mena city is considered, which is divided into several subareas. The PSO and KTS algorithms are invoked to find the efficient placement of a single UAV for providing wireless coverage for outdoor and indoor users in a rectangle subarea, with the dimensions of 300 m × 150 m. More specifically, for each subarea, it is assumed that 35% of the total number of receivers inside the subarea is active users.

The simulations and analysis of the simulation results are performed using MATLAB ^®^. Moreover, all simulation parameters are listed in Table 2.

In this scenario, it is considered that there are two buildings in the subarea, and each building consists of 12 floors. The number of outdoor receivers is 2750, where it is assumed that 35% of the total number of receivers is active users. On the other hand, three different numbers of indoor receivers, namely 600, 750 and 960, are considered. For the case of having 960 indoor users, it is assumed that there are 40 active receivers on each floor. Both outdoor and indoor receivers are non-uniformly distributed based on the beta random distribution function, f(x,y). The total number of active outdoor and indoor receivers is presented in Table 3. For each receiver, the data rate *r* is 0.5 Mbps.

**Figure 2:**
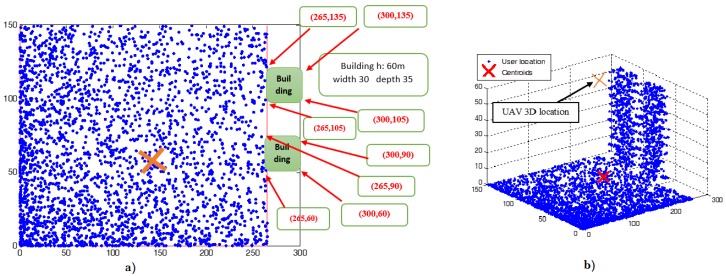
(**a**) Top view of the receivers’ distribution and the two buildings’ locations; (**b**) 3D view of outdoor and indoor receivers.

Figure 2a illustrates the distribution of the outdoor receivers and the coordinates of the two buildings inside the subarea; whilst Figure 2b illustrates the 3D view of the distribution of the indoor and outdoor receivers.

**Figure 3:**
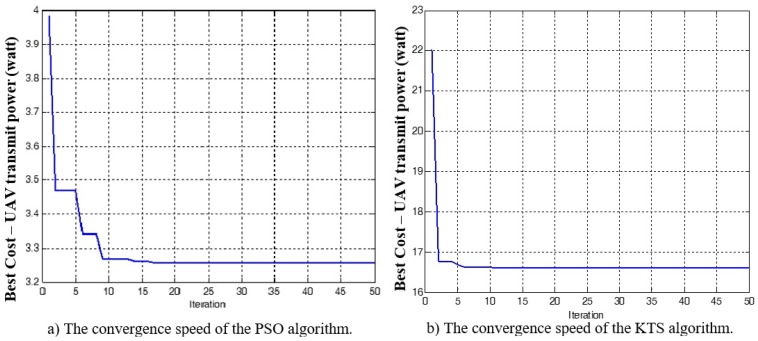
(**a**) The convergence speed of the PSO algorithm; (**b**) the convergence speed of the KTS algorithm, for the case of 960 indoor receivers inside each building.

The simulation results of the proposed algorithms, when varying the number of indoor active users, are shown in Table 3. More specifically, this table compares the minimum required total UAV transmit power that satisfies the data rate of all receivers, when invoking the PSO and KTS algorithms. The results show that the proposed algorithm using PSO requires less transmit power when the UAV is at the 3D efficient location; more specifically, for the case of having the greatest number of indoor receivers, 960. The 3D location of UAV using the PSO algorithm is (161.66,62.79,62.12), as shown in Figure 2a. This requires a total UAV transmit power of 3.255 watts, as illustrated in Figure 3. On the other hand, the 3D location of UAV using the KTS algorithm is (162.64,63.23,41.11), and the required UAV transmit power is 16.594 watts, as we can see in Figure 2b. Thus, the proposed algorithm using PSO exhibits about five-times improvement in terms of minimum required transmit power, when compared with that using the KTS algorithm.

This is because, in PSO, the efficient 3D UAV placement is determined without dividing the problem into subproblems. On the other hand, in KTS, the solution to find an efficient 3D placement of UAV is divided into two stages of solving two subproblems. In the first subproblem, K-means is used to find the 2D placement of UAV ($x_{UAV}$, $y_{UAV}$). Then, in the second subproblem, the ternary search algorithm is used to find the altitude ($z_{UAV}$) of UAV.

### 4.2. Providing Wireless Coverage Using Multiple UAVs

This section presents the simulation results of the proposed algorithms for the efficient deployment strategy of multiple UAVs equipped with directional antennas, to provide wireless coverage for receivers distributed over a large area of a square, rectangle and circle. The simulations and analysis of the simulation results are performed using MATLAB^®^.

#### 4.2.1. Case of a Square Region

**Figure 4:**
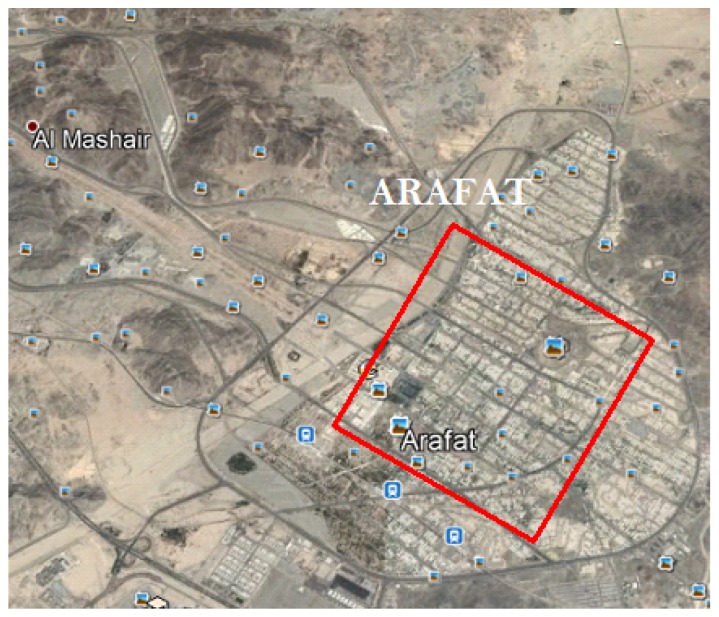
Square coverage area of 2 km × 2 km.

In this scenario, the case of providing wireless coverage for gatherings of pilgrims in Arafat city that forms a square shape is considered, as shown in Figure 4. The square region has dimensions of 2 km × 2 km.

In this simulation, the CPT and the results in Table 1 are utilized to find the maximum radius of *n* identical circles that can be packed inside the targeted coverage area of a square shape. Each circle is referred to as a subarea. Then, the 3D placement for UAVs that minimizes the total UAV transmit power required to provide wireless coverage for all outdoor receivers within the circle subarea is determined. For each circle cell, it is assumed that 35% of the total number of receivers inside the coverage circle is active.

All simulation parameters are listed in Table 2.

Table 4 presents the simulation results of the proposed algorithms to pack *n* equal circles inside a 2 km × 2 km square region, for *n* = 8 to *n* = 22. More specifically, this table presents the maximum radius of the circle, *r*, such that the coverage area and density are maximized for each case of packing *n* identical circles in the square region. Consequently, the corresponding optimal 3D placement of UAVs that minimizes the total transmit power required to provide wireless coverage for all receivers inside the circle is presented in Table 4. The center coordinate of the circle cell is denoted as $\left(x_{i},y_{i}\right)$, which refers to UAV 2D location, and $z_{i}$ is the optimal UAV altitude. The maximum packing density of the coverage region is also evaluated. The directional antenna half beamwidth $\theta_{i}/2$ is defined as $\tan{}^{-1}\left(r_{i}/h_{i}\right)$, where $r_{i}$ is the radius of circle *i* and $h_{i}$ is the optimal UAV altitude.

It can be seen from Table 4 that the highest achievable density of the packed circles in a square occurs when n=9 and n=16, when the proposed algorithm is invoked. However, the minimum UAV transmit power when packing n=9 circles is very high.

**Figure 5:**
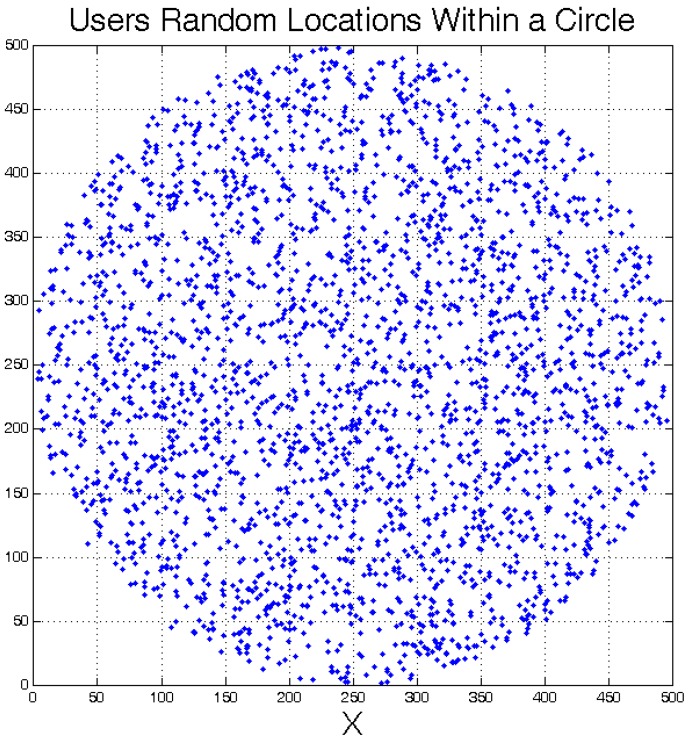
Top view of uniformly distributed active users inside the circle subarea with a radius of 250 m, when n=16.

**Figure 6:**
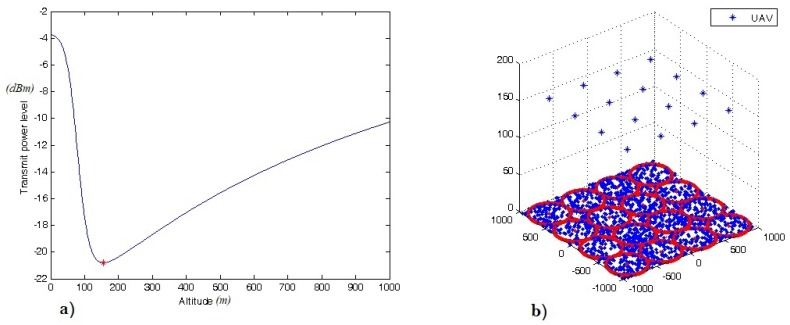
(**a**) The optimal UAV altitude is at 156 m; (**b**) the optimal placement of 16 UAVs with a circle radius of 250 m, when n=16.

For the case of packing n=16 circles, the maximum circle radius *r* is 250 m. For this case, the distribution of 2745 active receivers inside the subarea is illustrated in Figure 5. It can be seen from Figure 6a that the corresponding optimal UAV altitude is at 156 m, when the minimum transmit power is at the minimum.

Thus, the distribution of the outdoor receivers and the corresponding optimal 3D placement of 16 UAVs can be illustrated as shown in Figure 6b. Each UAV provides wireless coverage for one circle cell (subarea) with a radius of 250 m. The center of each subarea ($x_{i}$,$y_{i}$) ϵ*i* = 1, …, *n* is the $\left(x_{UAV},y_{UAV}\right)$ location, whilst the optimal altitude of all UAVs is at $z_{UAV}$ = 156 m. At this location, the UAV required transmit power for each UAV${}_{i}$ϵ1,…,16 is equal to 20 mwatt, and the maximum coverage density $d_{n}$ is 78.5%.

**Figure 7:**
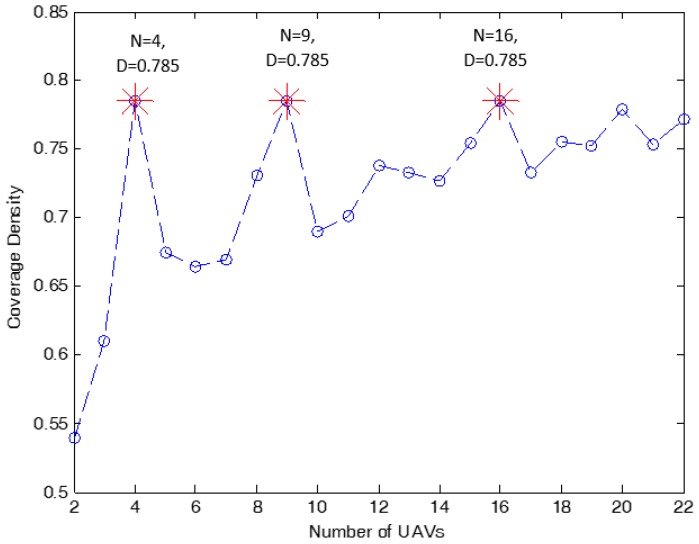
The coverage density for n=2 to n=22 identical circles inside a square coverage area with the dimensions of 2 km × 2 km.

Specifically, the optimal 3D locations (coordinates) of 16 UAVs are: (−750,−750,156); (−250,−750,156); (250,−750,156); (750,−750,156); (−750,−250,156); (−250,−250,156); (250,−250,156); (750,−250,156); (−750,250,156); (−250,250,156); (250,250,156); (750,250,156); (−750,750,156); (−250,750,156); (250,750,156); (750,750,156). On the other hand, Figure 7 presents the square coverage density for different *n* values. In this figure, we can see that when n=4,9,16, the maximum coverage density occurs where $d_{n}$ = 0.785. However, the lowest UAV required transmit power occurs when n=16. Hence, the UAV optimal altitude is at $z_{UAV}$ = 156 m.

#### 4.2.2. Case of a Rectangle Region

**Figure 8:**
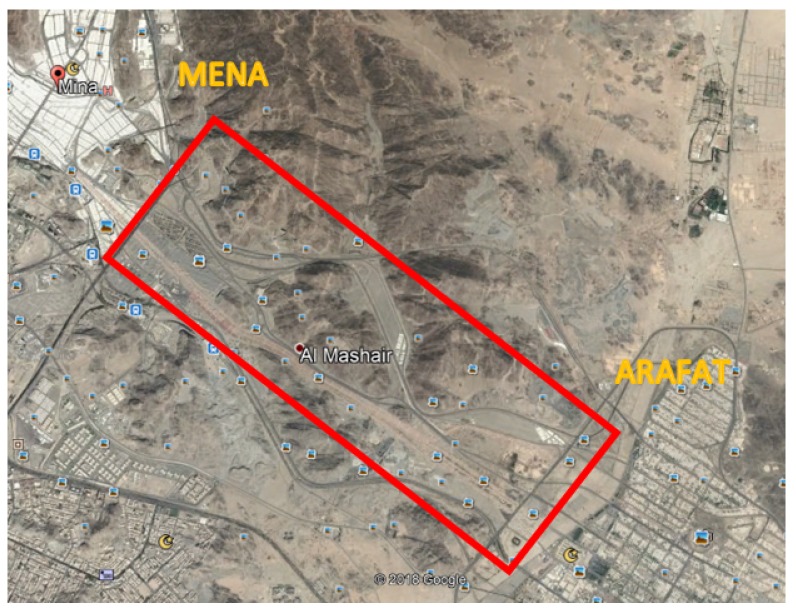
Rectangle coverage area with the dimensions of 6 km × 1.8 km.

In this scenario, the case of providing wireless coverage for gatherings of pilgrims that forms a rectangle shape is considered, which is located in the area between Arafat city and Mena city, as shown in Figure 8. The rectangle region has the dimensions of 6 km × 1.8 km. More specifically, this simulation finds the maximum radius of *n* identical non-overlapping circles that can be packed inside the targeted coverage area of a rectangle shape. Similar to the case of packing circles into a square region, each circle is referred to as a subarea. Then, for each subarea, the optimal 3D UAV placement is determined. The objective is to minimize the total transmit power required to provide wireless coverage for all receivers inside the circle cell, such that the packing density is maximized. The coverage area contains roads and paths for users’ movement, and there are areas where nobody crosses. Therefore, for each circle cell, it is assumed that 25% of the total number of users inside the coverage circle is active.

Table 5 presents the simulation results for packing *n* identical circles inside a 6 km × 1.8 km rectangular region, for n=10 to n=33. The 3D placement of UAV that minimizes the total transmit power required to cover all active users in each circle cell is determined. The center coordinate of the circle cell is denoted as $\left(x_{i},y_{i}\right)$, which refers to UAV 2D location, and $z_{i}$ is the optimal UAV altitude. The maximum packing density of the coverage region and the antenna half beamwidth for each cell is also evaluated. It can be seen from Table 5 that the highest achievable density of the packed circles in a rectangle occurs when n=26 and the proposed algorithm is invoked.

**Figure 9:**
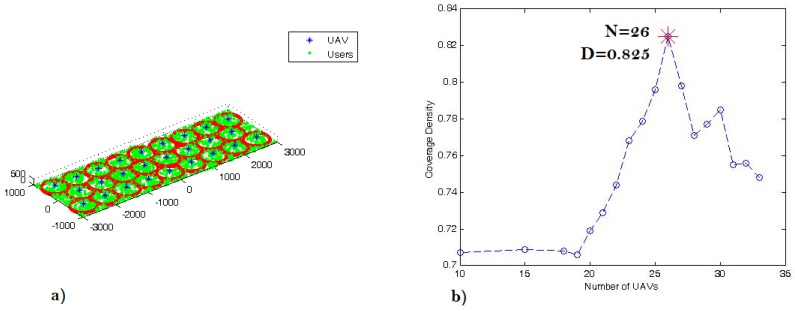
(**a**) Top 3D view of the users distribution and the UAVs’ placement inside the coverage area 6 km × 1.8 km; (**b**) the coverage density for packing *n* identical circles inside the coverage area.

Figure 9a illustrates the distribution of the outdoor receivers and the corresponding optimal 3D placement of 26 UAVs. Each UAV provides wireless coverage for one circle cell (subarea) with a radius of 330 m. The center of each subarea ($x_{i}$,$y_{i}$) ϵ*i* = 1, *…*, *n* is the $\left(x_{UAV},y_{UAV}\right)$ location, whilst the optimal altitude of all UAVs is at $z_{UAV}$ = 206 m. At this location, the UAV required transmit power for each UAV${}_{i}$ϵ1,…,26 is equal to 0.246 watts, and the maximum coverage density $d_{n}$ is 82.5%. More specifically, the efficient 3D placements (coordinates) of 26 UAVs that maximizes the coverage area and density are: (−2670,−570,206); (−2002,−570,206); (−1335,−570,206); (−667,−570,206); (0,−570,206); (667,−570,206); (1335,−570,206); (2002,−570,206); (2670,−570,206); (−2336,0,206); (−1669,0,206); (−1001,0,206); (−334,0,206); (334,0,206); (1001,0,206); (1669,0,206); (2336,0,206); (−2670,570,206); (−2002,570,206); (−1335,570,206); (−667,570,206); (0,570,206); (667,570,206); (1335,570,206); (2002,570,206); (2670,570,206). Moreover, for the case of packing n=26 circles, the coverage density is 82.5%, as shown in Figure 9b, and each UAV requires 0.246 watts to cover all active receivers inside the cell with a radius of *r* = 330 m.

#### 4.2.3. Case of a Circular Region

**Figure 10:**
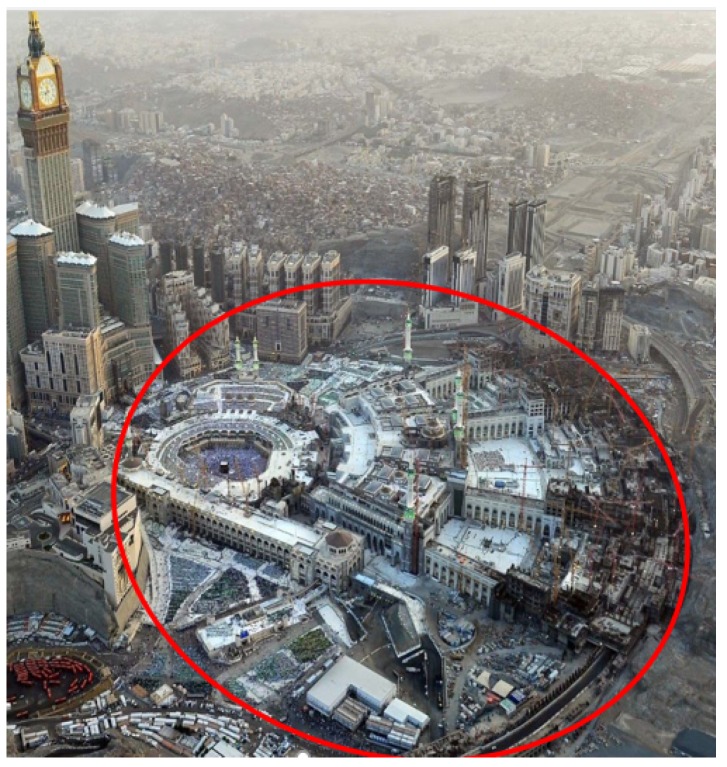
Circular coverage region with *r* = 1125 m.

In this scenario, the case of providing wireless coverage for gatherings of pilgrims in Masjid Al-Haram and its outer courtyard is considered, which forms a circular shape, with a radius of *r* = 1.125 km, as shown in Figure 10. This region is divided into identical circles, and each circle (subarea) is served by one UAV. This simulation is performed to find the maximum radius of *n* identical non-overlapping circles that can be packed inside the targeted area of the circular region. Then, the 3D UAV placement that minimizes the total transmit power required to provide wireless coverage for active receivers inside the circle cell for each circle is determined. For each circle cell, it is assumed that 35% of the total number of receivers inside the circular region is active.

The simulation results for packing *n* identical circles inside a circular region with *r* = 1.125 km are presented in Table 6. Then, the efficient 3D placement of the UAV that minimizes the total transmit power required to cover all active users inside the circle cell (subarea) for each circle cell is found. The center coordinate of the circle cell is denoted as $\left(x_{i},y_{i}\right)$, which refers to UAV 2D location, and $z_{i}$ is the optimal UAV altitude. The maximum packing density of the coverage region and the antenna half beamwidth for each case of packing *n* identical circles is also evaluated.

**Figure 11:**
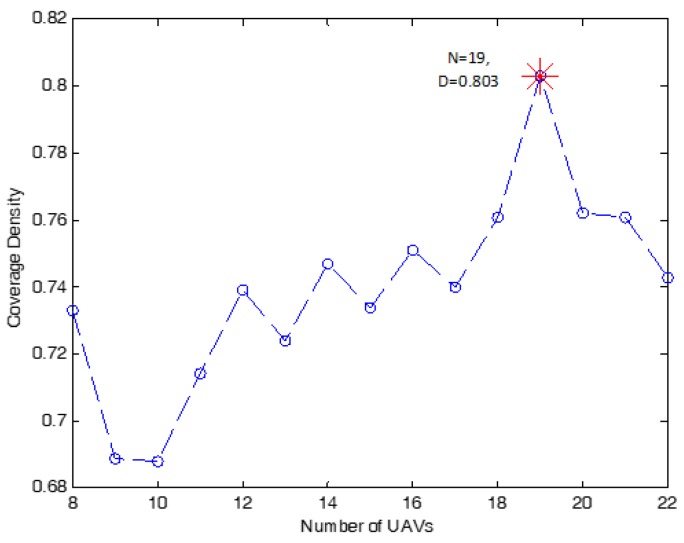
The coverage density of the circular coverage region with *r* =1125 m.

It can be seen from Figure 11 that the best coverage density is 80.3% when n=19. For the case of packing n=19 circles in the circular region, it can be observed from Table 6 that the required transmit power is 13 mwatt for each UAV to provide coverage for all active receivers inside the circle cell with a radius of *r* = 231 m; whilst the optimal 3D placement of 19 UAVs is at 144 m.

### 4.3. Discussion

In this section, the main observations obtained from the simulation results presented in Section 4.1 and Section 4.2 are discussed.

It is observed that the UAV required transmit power depends on the number of active receivers within the coverage subarea and, hence, affects the altitude of the UAV. More specifically, in the case of finding the efficient 3D placement of a single UAV in providing wireless coverage for outdoor and indoor users, it is observed that as the number of indoor active users increases, the required transmit power increases, which causes the altitude of the UAV to increase, as presented in Table 3. Similar performance is observed for the case of providing wireless coverage using multiple UAVs. More specifically, for this case, as the number of circle cells (subareas) increases, the number of users inside each subarea decreases. Hence, the UAV required transmit power decreases, and consequently, the altitude of the UAV decreases, as observed in Table 4, Table 5 and Table 6.

With the specific observation from the case of providing wireless coverage using a single UAV, the problem is formulated to find the efficient 3D placement with the objective to minimize the UAV transmit power. In this case, the choice of the algorithm used to find the efficient 3D placement affects the UAV required transmit power. More specifically, it was observed that the PSO algorithm performed better than the KTS algorithm to find the 3D efficient placement of the UAV, where the 3D location found using the PSO algorithm requires less transmit power, by a factor of 1/5 compared to that when using the KTS algorithm, as discussed in Section 4.1.

Providing wireless coverage for outdoor users within a larger coverage area requires more than one UAV. Thus, it is proposed to utilize CPT in dividing the coverage area into subareas, such that the coverage area and density are maximized. Then, the optimal UAV altitude is determined with the objective of minimizing the UAV required transmit power. It is observed that the large number of UAVs does not necessarily results in the maximum coverage density.

As observed in Table 4, the maximum coverage density is obtained when the number of identical cells n=4,9 and 16 for the case of providing wireless coverage for users in a square region with the dimensions of 2 km × 2 km. However, the UAV required transmit power is very high when n=4,9. Thus, for this case, the deployment of 16 UAVs is capable of providing the maximum coverage density of 78.5%, with the optimal altitude of all UAVs at $z_{UAV}$ = 156 m. At this location, the required transmit power for each UAV is equal to 20 mwatt, which is not the minimum. The minimum transmit power of 0.124 mwatt is observed when n=22, which is when the number of active users within the subarea is the smallest.

A similar observation is obtained from Table 5 and Table 6, where there is a trade-off between the maximum coverage density and the UAV required transmit power. More specifically, having the maximum coverage density does not guarantee having the smallest value of the UAV required transmit power. However, the required transmit power is reasonably small.

## 5. Conclusions

In this paper, a UAV deployment strategy is proposed, for providing wireless coverage for users in small and large coverage areas of massively crowded events. More specifically, in a small coverage area, the problem was formulated to provide wireless coverage for outdoor and indoor users, with the objective to minimize the UAV transmit power. In this case, the PSO and KTS algorithms were used to find an efficient 3D UAV placement that minimizes the total required transmit power and satisfies the data rate for users. It was observed that the PSO algorithm performed better than the KTS algorithm to find the 3D efficient placement of UAV. More specifically, for a single UAV at the 3D location found using the PSO algorithm, the UAV requires less transmit power, by a factor of 1/5 compared to that when using the KTS algorithm.

For the deployment of UAV in providing wireless coverage for users in a large area of massively crowded events, CPT is utilized. More specifically, in a large coverage area, CPT is utilized to find the efficient 3D placements of multiple UAVs by packing identical, non-overlapping and interference free circle cells inside three different 2D shapes’ coverage area, namely square, rectangle and circular regions. The problems were formulated with the objective to maximize the total coverage area and coverage density. Then, the efficient altitude placement of each UAV was found using the formulation that minimizes the transmit power in each circle cell.

For the case of a square region, it was found that the deployment of 16 UAVs at the optimum altitude of 156 m is capable of providing 78.5% coverage density of a square region with the dimensions of 2 km × 2 km, where each UAV required 20 mwatt. For the case of a rectangle region, the deployment of 26 UAVs at the optimum altitude of 206 m is capable of providing 82.5% coverage density of a rectangle region with the dimensions of 6 km × 1.8 km, where each UAV requires 0.246 watt. For the case of a circular region, the deployment of 19 UAVs at the optimum altitude of 144 m is capable of providing 80.3% coverage density of the circular region with the radius of 1.125 km, where each UAV requires 13 mwatt. However, it was also observed that an increase of the number of UAVs did not necessarily result in the maximum coverage density.

As future work, it is proposed to generalize the solution techniques to provide wireless coverage using UAVs by utilizing the packing of identical circles inside convex and non-convex regions. Moreover, it is also proposed to study the presence of interference between congruent cells in providing wireless coverage.

## Figures and Tables

**Table 1:** Results of packing of *n* equal circles in a unit square.

*n*	$r_{n}$	$d_{n}$	Ref.	*n*	$r_{n}$	$d_{n}$	Ref.
2	0.292893	0.539	[32]	13	0.133994	0.733	[27]
3	0.254333	0.6096	[32]	14	0.128556	0.727	[27]
4	0.250000	0.785	[32]	15	0.126478	0.754	[27]
5	0.207107	0.674	[32]	16	0.125000	0.785	[27]
6	0.187681	0.664	[32]	17	0.117186	0.733	[27]
7	0.174458	0.669	[33]	18	0.115522	0.755	[27]
8	0.170541	0.731	[33]	19	0.112265	0.752	[27]
9	0.166666	0.785	[34]	20	0.111382	0.779	[27]
10	0.148204	0.690	[27]	21	0.106839	0.753	[27]
11	0.142399	0.701	[27]	22	0.105665	0.772	[27]
12	0.139959	0.738	[27]				

**Table 2:** Simulation parameters.

Parameter	Value	Parameter	Value
Carrier frequency $\left(f_{c}\right)$	2 GHz	(Vmin, Vmax, Vsize)	(0, 1000, 3)
Noise power (Np)	−120 dBm	Population size (N_pop)	50
Total available	50 MHz	Max number of iterations	50
bandwidth		(N_it)	
UAV transmit power	$P_{t}$ = 5 watt	(κ, $\phi_{1}$, $\phi_{2}$)	(1, 2.05, 2.05)
Data rate (r)	0.5 Mbps	Tolerance (ϵ)	0.1
**Environment Parameter**	**Value**	**Environment Parameter**	**Value**
*a*	9.6	$\eta_{LSO}$	1
*b*	0.28	$\eta_{NLSO}$	20

**Table 3:** Simulation results for providing wireless coverage using a single UAV for outdoor and indoor receivers. KTS, K-means with Ternary Search.

Algorithm	(Outdoor) Subarea Dimensions	(Indoor) Building Dimensions	Number of Outdoor Users	Number of Active Indoor Users	Efficient UAV Placement ($x_{\mathrm{UAV}}$, $y_{\mathrm{UAV}}$, $z_{\mathrm{UAV}}$)	UAV Transmit Power (watt)	Enhanced PSO than KTS
PSO	300 m × 150 m	(35,30,60)	2750	One Building, 12 floors → 600	(117.49, 58.78, 60.89)	0.42	5.7×
KTS					(129.53, 60.26, 49.1)	2.39	
PSO	300 m × 150 m	(35,30,60)	2750	One Building, 12 floors → 750	(151.95, 58.3, 61.5)	0.86	5.4×
KTS					(158, 60.76, 46.5)	4.639	
PSO	300 m × 150 m	(35,30,60)	2750	Two Buildings, 12 floors →960	(161.66, 62.79, 62.12)	3.255	5.1×
KTS					(162.64, 63.23, 41.11)	16.594	

**Table 4:** Simulation results for packing *n* circles in a 2 km × 2 km square region ($x_{i}$,$y_{i}$) ϵ I = 1, *…*, *n*.

*n*	Circle Radius *r*	Number of Active Receivers 35%	Optimal 3D UAV Placement	UAV Transmit (Power) watt	Density	Antenna Half Beamwidth *θ*/2
8	341.1 m	5076	($x_{i}$, $y_{i}$, 213)	5095 Very High	0.731	58.01
9	333.3 m	4925	($x_{i}$, $y_{i}$, 208)	2068 Very High	0.785	58.03
10	296.4 m	3819	($x_{i}$, $y_{i}$, 185)	16.5	0.690	58.03
11	284.8 m	3565	($x_{i}$, $y_{i}$, 178)	3.39	0.701	58.0
12	279.9 m	3445	($x_{i}$, $y_{i}$, 175)	1.61	0.738	57.9
13	267.9 m	3200	($x_{i}$, $y_{i}$, 167)	0.373	0.733	58.06
14	257.1 m	2973	($x_{i}$, $y_{i}$, 160)	0.091	0.727	58.1
15	252.9 m	2860	($x_{i}$, $y_{i}$, 158)	0.043	0.754	58.0
16	250.0 m	2745	($x_{i}$, $y_{i}$, 156)	0.020	0.785	58.03
17	234.4 m	2422	($x_{i}$, $y_{i}$, 147)	2.60 $\times 10^{-3}$	0.733	57.91
18	231.0 m	2328	($x_{i}$, $y_{i}$, 144)	1.40 $\times 10^{-3}$	0.755	58.06
19	224.5 m	2226	($x_{i}$, $y_{i}$, 140)	7.34 $\times 10^{-4}$	0.752	58.05
20	222.8 m	2136	($x_{i}$, $y_{i}$, 139)	3.87 $\times 10^{-4}$	0.779	58.04
21	213.7 m	2025	($x_{i}$, $y_{i}$, 133)	2.03 $\times 10^{-4}$	0.753	58.10
22	211.3 m	1953	($x_{i}$, $y_{i}$, 132)	1.24 $\times 10^{-4}$	0.772	58.0

**Table 5:** Simulation results for packing *n* circles in a 6 km × 1.8 km rectangle ($x_{i}$,$y_{i}$) ϵ i = (1, *…*, *n*).

*n*	Circle Radius *r*	Number of Active Receivers 35%	Optimal 3D UAV Placement	UAV Transmit (Power) watt	Density	Antenna Half Beamwidth *θ*/2
10	493 m	7634	($x_{i}$, $y_{i}$, 307)	Very High	0.707	58.089
15	402 m	5020	($x_{i}$, $y_{i}$, 251)	Very High	0.709	58.02
18	368 m	4181	($x_{i}$, $y_{i}$, 229)	53.9 High	0.708	58.107
19	357 m	3953	($x_{i}$, $y_{i}$, 223)	11.95	0.706	58.009
20	351 m	3843	($x_{i}$, $y_{i}$, 219)	6.088	0.719	58.039
21	345 m	3736	($x_{i}$, $y_{i}$, 215)	3.306	0.729	58.069
22	341 m	3626	($x_{i}$, $y_{i}$, 213)	1.59	0.744	58.010
23	339 m	3559	($x_{i}$, $y_{i}$, 211)	1.013	0.768	58.101
24	334 m	3518	($x_{i}$, $y_{i}$, 208)	0.847	0.779	58.087
25	331 m	3418	($x_{i}$, $y_{i}$, 206)	0.435	0.796	58.104
26	330 m	3384	($x_{i}$, $y_{i}$, 20 6)	0.246	0.825	58.026
27	319 m	3213	($x_{i}$, $y_{i}$, 199)	0.125	0.798	58.043
28	308 m	3006	($x_{i}$, $y_{i}$, 192)	0.036	0.771	58.062
29	303 m	2890	($x_{i}$, $y_{i}$, 189)	0.018	0.777	58.046
30	300 m	2821	($x_{i}$, $y_{i}$, 187)	0.011	0.785	58.063
31	289 m	2636	($x_{i}$, $y_{i}$, 180)	0.0037	0.755	58.084
32	285 m	2543	($x_{i}$, $y_{i}$, 178)	0.0020	0.756	58.013
33	279 m	2459	($x_{i}$, $y_{i}$, 174)	0.0012	0.748	58.050

**Table 6:** Simulation results for packing *n* identical circles in a circle region with *r* = 1.125 km, ($x_{i}$, $y_{i}$) ϵ*i* = 1, *…*, *n*.

*n*	Radius (*r*) (Unit Circle)	Circle Radius (*r*) (*R* = 1125 m)	Number of Active Receivers 35%	Optimal 3D UAV Placement	UAV Transmit (Power) watt	Density	Antenna Half Beamwidth *θ*/2
8	0.30259339	340	5077	($x_{i}$, $z_{i}$, 212)	53,564 Very High	0.7325020	58.06
9	0.27676865	311	4252	($x_{i}$, $y_{i}$, 194)	267.1 Very high	0.68940799	58.04
10	0.26225892	295	3820	($x_{i}$, $y_{i}$, 184)	17.43	0.68779743	58.05
11	0.2548547	287	3600	($x_{i}$, $y_{i}$, 179)	4.29	0.71446011	58.05
12	0.24816347	279	3445	($x_{i}$, $y_{i}$, 174)	1.63	0.7390213	58.05
13	0.23606798	266	3082	($x_{i}$, $y_{i}$, 166)	0.166	0.72446517	58.03
14	0.23103073	260	2973	($x_{i}$, $y_{i}$, 162)	0.0874	0.74725276	58.07
15	0.22117254	249	2746	($x_{i}$, $y_{i}$, 155)	0.0199	0.73375938	58.10
16	0.21666474	244	2634	($x_{i}$, $y_{i}$, 152)	9.80 $\times 10^{-3}$	0.75109777	58.08
17	0.20867967	235	2422	($x_{i}$, $y_{i}$, 147)	2.40 $\times 10^{-3}$	0.74030245	57.97
18	0.20560465	231	2318	($x_{i}$, $y_{i}$, 144)	1.30 $\times 10^{-4}$	0.76091887	58.06
19	0.20560465	231	2318	($x_{i}$, $y_{i}$, 144)	1.30 $\times 10^{-4}$	0.80319214	58.06
20	0.19522401	220	2127	($x_{i}$, $y_{i}$, 137)	3.76 $\times 10^{-4}$	0.76224829	58.09
21	0.19039215	214	2026	($x_{i}$, $y_{i}$, 133)	2.01 $\times 10^{-4}$	0.76123256	58.14
22	0.18383303	207	1851	($x_{i}$, $y_{i}$, 129)	6.29 $\times 10^{-5}$	0.7434808	58.07
